# An Early Crusade against Criminal Abortion

**Published:** 1912-08

**Authors:** 


### An Early Crusade Against Criminal Abortion

In September, 1858, the *Buffalo Medical Journal* called attention to this evil, and called upon the profession and the lay press to cooperate in a campaign. The advertisement of regulators in religious papers was emphatically denounced. A spirit of cooperation was evidently aroused and several letters on the subject and additional editorial articles were printed. One physician reported a peculiarly harrowing case of a widow who importuned him to get her out of trouble. He refused but was called, in the course of a few weeks to attend her. She had taken some abortifacient whose nature she refused to disclose, had given birth to a living though premature infant and had burned it alive.

This is only one of many matters demanding professional attention that have illustrated the progressive spirit of the western New York physicians, as voiced in their JOURNAL.

---

WHAT MAY HAPPEN. "We are afraid that we shall have to dun in our next issue. It will be only dire necessity that will drive us to the alternative of asking for our due, therefore we beg those indebted to us to communicate.........." The foregoing was printed in the *Buffalo Medical Journal* of August, 1858. The present editor is very glad to state that there are comparatively few delinquent subscribers. Bills are inclosed in the present issue and we would appreciate a prompt response, not so much for obvious reasons as that attention to subscription receipts at the beginning of the year and en masse, simplifies bookkeeping, allows valuable times to be devoted to matters of greater interest to readers, instead of to sordid business details, strengthens our position with the postoffice and with advertisers. Any errors in bills, addresses, etc., should be reported immediately.

